# A global systematic review of forest management institutions: towards a new research agenda

**DOI:** 10.1007/s10980-022-01577-8

**Published:** 2022-12-24

**Authors:** Jude Ndzifon Kimengsi, Raphael Owusu, Shambhu Charmakar, Gordon Manu, Lukas Giessen

**Affiliations:** 1 Forest Institutions and International Development (FIID) Research Group, Chair of Tropical and International Forestry, Faculty of Environmental Sciences, Technische Universität Dresden, Dresden, Germany; 2 Department of Geography, The University of Bamenda, Bamenda, Cameroon; 3 Food and Agricultural Organization (FAO), Rome, Italy; 4 Chair of Tropical and International Forestry, Faculty of Environmental Sciences, Technische Universität Dresden, Dresden, Germany

**Keywords:** Rules, Customs, Compliance, Forest resources, Access, Methods, Outcomes

## Abstract

**Context:**

Globally, forest landscapes are rapidly transforming, with the role of institutions as mediators in their use and management constantly appearing in the literature. However, global comparative reviews to enhance comprehension of how forest management institutions (FMIs) are conceptualized, and the varying determinants of compliance, are lacking. And so too, is there knowledge fragmentation on the methodological approaches which have and should be prioritized in the *new research agenda* on FMIs.

**Objectives:**

We review the regional variations in the conceptualization of FMIs, analyze the determinants of compliance with FMIs, and assess the methodological gaps applied in the study of FMIs.

**Methods:**

A systematic review of 197 empirically conducted studies (491 cases) on FMIs was performed, including a directed content analysis.

**Results:**

*First,* FMIs literature is growing; multi-case and multi-country studies characterize Europe/North America, Africa and Latin America, over Asia. *Second*, the structure-process conceptualization of FMIs predominates in Asia and Africa. *Third*, global south regions report high cases of compliance with informal FMIs, while non-compliance was registered for Europe/North America in the formal domain. *Finally, m*ixed-methods approaches have been least employed in the studies so far; while the use of only qualitative methods increased over time, the adoption of only quantitative approaches witnessed a decrease.

**Conclusion:**

Future research should empirically ground informality in the institutional set-up of Australia while also valorizing mixed-methods research globally. Crucially, future research should consider multidisciplinary and transdisciplinary approaches to explore the actor and power dimensions of forest management institutions.

**Supplementary Information:**

The online version contains supplementary material available at 10.1007/s10980-022-01577-8.

## Introduction

Globally, forests are at a crossroads—characterized by rapid transformation (Garcia et al. 2020). For instance, Global Forest Watch estimated that forest loss around the globe reached 29.7 million hectares as of 2016, indicating a 51% increase since 2015. For tropical forests, the loss was estimated at 12 million hectares (the size of Belgium) in 2018 (Weisse and Goldman 2017; Garcia et al. 2020). While such changes are linked to natural (e.g., climate change) and human-induced drivers such as land-use change (Rounsevell et al. 2006; Meyfroidt and Lambin 2011; Aguiar et al. 2016; Houghton and Nassikas 2018), they form part of a complex transformation system—mediated by socio-economic, political, and institutional forces (Malhi et al. 2014). This validates the role of institutions as a key enhancing or constraining factor in determining forest resources access, use and management (Cleaver 2017). Institutions are viewed as highly abstract and invisible conditions in the political environment. They constitute cognitive, normative, and regulatory structures which provide stability and meaning to social behaviour. Institutions are carried across multiple vehicles, including cultures, structures and routines, operating at multiple levels of jurisdiction (Scott 1995). The transformation of forest landscapes at various timescales is characterized by net tropical forest loss (Geist and Lambin 2002; Kissinger et al. 2012; Song et al. 2018). Furthermore, regional variations in the drivers (Curtis et al. 2018) exist: in Latin America, transformations are largely rooted in ranching and soybean expansion (Rudel et al. 2009; Verburg et al. 2014; Tyukavina et al. 2017), while subsistence agriculture drives the transformation process in Africa (Hosonuma et al. 2012; Tyukavina et al. 2018). Transformations in Asia are significantly linked to industrial processes and small-holder farming (Rudel et al. 2009; Turubanova et al. 2018).

While forests are declining (Weisse and Goldman 2017), their roles in the resolution of global socio-ecological challenges (e.g., climate change mitigation and poverty reduction) remain unrivalled (Oldekop et al. 2020; Nerfa et al. 2020). Scholars submit that governance mechanisms, especially the role of institutions, remain primordial in shaping forests access, use, and management. For this reason, institutions—the rules of the game—continually gain relevance (Agrawal and Gupta 2005; Dixon and Wood 2007; Kimengsi et al. 2021). Variations exist in the way institutions are conceptualized. For instance, following the structure process dichotomy (Fleetwood 2008a, b), institutions relate to tissues of social relations linking groups and communities (structures) and a set of rules, conventions and values, among others (processes) (Fleetwood 2008a; Bernardi et al. 2007). It is, however, difficult to provide a dividing line between the processes and structures; processes (rules) guide the formation of structures, while structures, on the other hand, oversee and enforce rules (Fleetwood 2008a; Ntuli et al. 2021). However, structures differ from processes in terms of their functioning; structures could represent forest management organizations as an entity, and not the rules (processes) which they produce (Ntuli et al. 2021). Both structures and processes are subjected to a categorization as either formal (written and codified laws, largely state driven) and informal (unwritten or uncodified rules that transcend generations) (Osei-Tutu et al*.* 2014; Yeboah-Assiamah et al. 2017). Furthermore, and on the basis of source, institutions could be categorized following the endogenous—exogenous dichotomy; the former relates to community-specific complex and embedded rules, while the latter denotes institutions introduced by the state and international agencies (Yeboah-Assiamah et al. 2017; Kimengsi et al. 2022a, b, c). By and large, these categories of institutions exist to provide order in the midst of ‘chaos’, with regards to the sustainable management of forest resources (Beunen and Patterson 2019).

While forest landscapes are transforming, institutions have also been subjected to several dimensions of change. Their evolution over time manifests through formation, reformation, disintegration, and modification in several contexts, including Africa (Haller et al. 2016; Friman 2020; Kimengsi et al. 2022a, b, c), Asia (Haapal and White 2018; Steenbergen and Warren 2018), and Latin America (Faggin and Behagel 2018; Gebara 2019). This brings to fore the notion of ephemeral, intermittent and perennial institutions (Kimengsi et al. 2021)—borrowed from the geographic classification of streams (Gomes et al. 2020). *Ephemeral* refers to short-term stream movements (institutional arrangements), *intermittent* is analogous to medium-term/seasonal streams (medium-term institutional arrangements), and *perennial* relates to streams that flow all through—analogous to more long-term, enduring institutions (Kimengsi et al. 2021). Therefore, the search for perennial (enduring) institutions is top on the scientific and policy agenda (Ostrom 1990; Kimengsi et al. 2021). This is important to support the attainment of objectives such as halting forest loss and improving forest cover and species diversity (Bare et al. 2015; Assa 2018), sustaining livelihoods and economic welfare (Buchenrieder and Balgah 2013; Foundjem-Tita et al. 2018), and engendering equity and fairness in the distribution of proceeds from forest systems (Faye et al. 2017). However, studies on institutions and institutional change are seemingly at an impasse; it seems difficult to proceed with the framing of forward-looking research questions linked to forest management institutions (FMIs). The impasse is rooted in the largely fragmented and unstructured institutional analysis around forest settings that harbor conflicts linked to emerging and persistent resource use inequalities (Gautam et al. 2004; Soliev et al. 2021). Additionally, the multiplicity of institutional variables and the lack of a consensus on which of the methods—qualitative or quantitative—is best suited for analyzing institutions and institutional change (Kimengsi et al. 2022a, b, c) further validate the need to surmount this impasse. In this regard, a systematic review of the global knowledge base on FMIs is imminent. Furthermore, details on the methods to prioritize in future studies further validates the need for a review. Consequently, we seek answers to the following questions: (1) *How have FMIs been conceptualized and analyzed globally?* (2) *How varied are the (non)compliance determinants and outcomes of FMIs?* (3) *How can we conceptually and methodologically advance research on FMIs?* To provide answers to these interrogations, we undertake a review of FMIs. The study is inspired by an earlier review conducted in the context of sub-Saharan Africa (Kimengsi et al. 2022b).

## Materials and methods

### Analytical framework

**Fig. 1:**
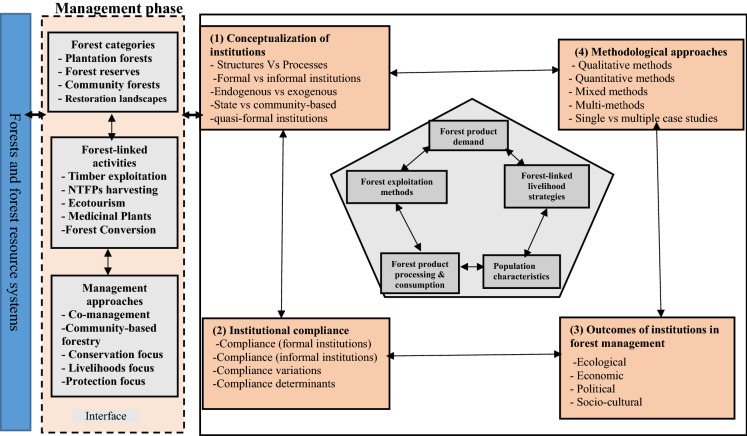
Analytical framework for the systematic review. Source: Based on Cleaver (2017), Haller et al. (2016), North (1990), Ostrom (1990, 2005), and Pretzsch et al. (2014). *NTFPs* Non-Timber Forest Product

In this review, we make use of the socio-ecological co-evolution framework (Pretzsch et al. 2014). The framework serves as a useful theoretical fundament to enhance understanding of the dynamics around forests and rural development. While allowing for the differentiation between humans and ecological subsystems, the framework also outlines the dynamic interactions between these two systems (Berkes et al. 1998; Pretzsch et al. 2014). The socio-ecological co-evolution framework is designed to enhance comprehension of the interactions between the social system (e.g., the community of forest users), the institutions that shape them, and the ecological system—forests. These interactions occur at the interface (management segment) of the framework. Besides providing a useful analytical lens to appreciate current levels of engagement in decision-making and the enforcement of institutional provisions, it also serves as a useful framework to understand how institutional change triggers the co-evolution of both ecological and social systems. The socio-ecological co-evolution framework is informed by the earlier works of Berkes et al. (1998), which bridged the hitherto divide between social research (centred around institutions), and ecological research, which emphasized cross-scale ecosystem dynamics. Worthy of note is the fact that other frameworks exist; for instance, the socio-ecological systems (SES) framework (Ostrom 2009) was proposed to explain complex systems involving resource systems (forests in this case), their resource units (e.g., timber), appropriators (e.g., timber exploiters), and governance systems (e.g., forest management rules) that continually interact to produce differential outcomes (Ostrom 2009). It explains that socio-ecological systems are constantly subjected to change. Some of these changes are rooted in institutions and institutional change processes (Rammel et al. 2007; Pretzsch et al. 2014). The socio-ecological co-evolution framework is employed for the following reasons: (1) with rapid transformations experienced in forest landscapes across the globe, scientific and policy circles need to extend their breadth of knowledge on how to further ‘marry’ social and ecological systems in forest management. (2) Institutional change is reflected through the decisions and actions of resource users at the interface of the framework. Therefore, understanding how these changes and their determinants precipitate (non)compliance is helpful in today’s dispensation, where forests are seen as crucial in stemming the upsurge of environmental crises. (3) The outcomes associated with the myriads of institutions need to be further appreciated to inform policy actors on the orientation of future FMIs. The socio-ecological co-evolution framework (Fig. 1) explains how changing societal demands and choices, influenced by the institutions in place, shape the type and magnitude of societal intervention in socio-ecological systems (e.g., forests).

The review, guided by the research questions, focuses on the management phase and the social segment of the socio-ecological co-evolution framework. The management phase represents an interface—a point where management decisions under different forest categories such as plantation forests, forest reserves, community forests and landscapes in want of restoration, are implemented. Institutions and institutional change processes drive such decisions. The management operations are construed as forest-linked activities which are informed by institutions regulating timber and NTFPs exploitation, ecotourism, medicinal plants’ extraction and forest conservation. Institutional arrangements in this socio-ecological system culminate in the derivation of different management approaches, such as co-management and community-based forestry with a focus on livelihoods and conservation. The social segment of the framework focuses on the conceptualization of forest management institutions (for instance, structures vs processes, formal vs informal, and endogenous vs exogenous). This segment also captured forest management institutional compliance with an emphasis on the variations and determinants. The segment on outcomes explored the ecological, economic, socio-cultural, and political outcomes of FMIs. The framework also has a segment which explores methodological approaches employed in the study of FMIs.

### Methodology

#### Data collection

The systematic review approach (Nightingale 2009a, b; Mengist et al. 2019) was employed in this study. Systematic reviews follow an established and standardized protocol for the search, appraisal and inclusion (or exclusion) of literature for subsequent analysis (Boell and Cecez-Kecmanovic 2015). This is different from the general review of literature which consists of a non-structured and highly subjective method of literature search and analysis (Kraus et al. 2020). The procedure was employed as follows: First a list of search terms (Appendix) was developed and used in the article search process. We targeted the following databases: Scopus, Science Direct, Google Scholar and Web of Science. Search terms such as forest management, forest governance, institutions, rules, norms, norms, laws, policies, community-based organizations, NGOs, associations, compliance, determinants, and outcomes were repeatedly employed in the search. The terms were combined with the respective regions (Africa, Asia, Australia, Europe/North America, Latin America), over a 15-year period (2006–2021). It should be noted that Europe and North America were clustered due to the observed similarity in their societal fabric and culture. We considered this timespan good enough to mirror contemporary evidence on the question of forest management institutions (FMIs). The search led to the initial identification of 920 articles. Four hundred thirty articles were identified from Web of Science, 104 from Google Scholar, 348 from Scopus and 38 from the Science Direct database. The search on Google Scholar did not produce a lot of articles. This is because grey literature was not considered during the search. Our emphasis was to derive literature which were published in internationally recognized databases. We then proceeded to deduplicate the articles—the deduplication process led to a reduction to 680 articles. Furthermore, article screening was performed with emphasis on the abstracts. This informed the decision to include or exclude the paper. In the selection, we targeted journal articles that were published in English and were empirically grounded. In cases where the abstract could not provide these details, we proceeded to review the methods and conclusions to inform inclusion (or exclusion). We excluded all grey literature during the article selection. This reduced the number of manuscripts to 197 (see Supplementary Excel Sheet), from which we derived 491 case studies; the cases were derived by considering the number of study areas that were included for analysis. We use ArcMap 10.5 to generate the map of the globe and the regions and/or countries where most of the case studies in this review paper were concentrated.

#### Data analysis

The articles retained were further read, and following the analytical framework (Fig. 1), a directed content analysis was performed (Hsieh and Shannon 2005). The directed content analysis began with a relevant theoretical framework—in this case, the socio-ecological coevolution framework. This framework provided a clear focus for the research questions under review. The key variables which were outlined in the framework (Fig. 1) informed the clustering of the data generated from the selected articles. For the selected articles, we read the abstract, methods and conclusion sections to generate data. The dataset was compiled in an excel sheet and further read; key texts which contained variables of interest were highlighted. These variables were then clustered following the established questions and themes for further analysis (Mayring 2000). Therefore, the highlighted texts, which contained data corresponding to the four thematic sections, were extracted from each article and organized under the main themes: *conceptualization of institutions, institutional compliance, outcomes of forest management institutions and methodological approaches*. We approached the conceptualization of institutions following the structure-process dimension (Fleetwood 2008a), the formal and informal dichotomy (North 1990), the endogenous vs exogenous institutional lens (Kimengsi et al. 2021) and the state vs community-based institutional dichotomy (Ntuli et al. 2021). Compliance denotes the extent to which forest users adhere to the institutional provisions in their communities. This translates to forest management outcomes which could be ecological, socio-economic and even political (Haller et al. 2016).

These were recorded in a Microsoft Excel sheet (Artmann and Sartison 2018). We considered this approach appropriate, considering that software extraction might ignore salient details owing to the complex nature of institutional variables. Besides narratives and content analysis, we used descriptive statistics to report the variations across the five regions. The descriptive analysis further aided in establishing institutional compliance and its determinants, the ecological, socio-cultural, economic, and political outcomes linked to forest management institutions, and the variations in methodological approaches employed.

## Results 

### Attributes of reviewed papers and case studies

**Fig. 2:**
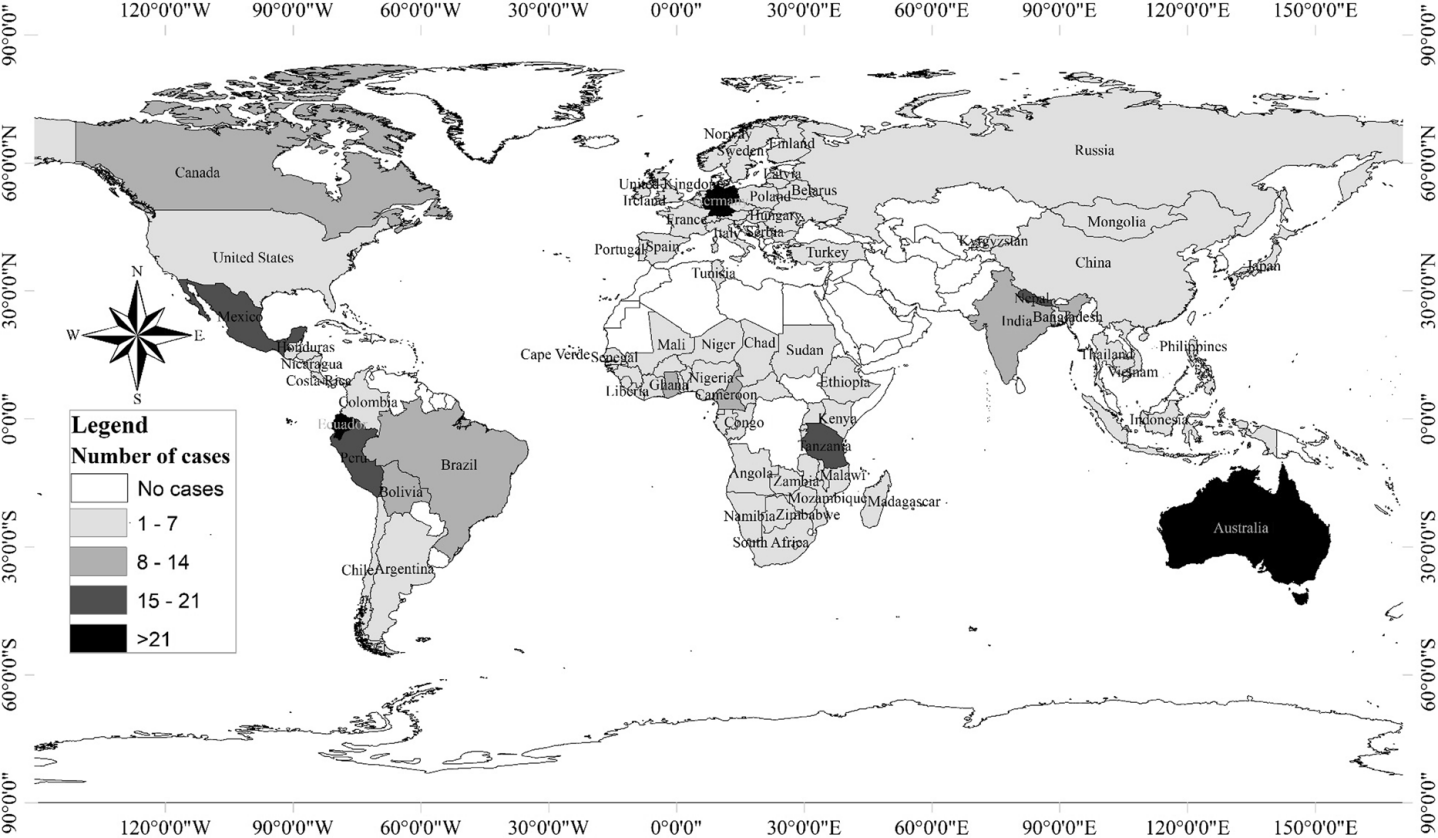
Spatial distribution of case studies on forest management institutions (2006–2021)

The review indicated that most of the articles emanate from Africa and Latin America—home to two of the world’s major forest ecosystems. This was followed by Asia. Case-wise, the study captured a total of 491 cases drawn from 99 countries across the globe (Fig. 2). The highest number of cases emanate from Europe/North America and Africa. This suggests that multi-case and multi-country studies have been significantly prioritized in these regions compared to single case/country studies for Asia and Australia.

In parts of Central Europe, studies to explore shifts towards new governance established that recent changes in institutional arrangements result from macro-political trends and the geopolitical strategy of some states (Sergent et al. 2018). In the United States and Canada, forest certification led to substantial changes in practices as enterprises embraced changes in forestry, environmental, social, and economic/system practices in the realm of forest certification (Moore et al. 2012). In the case of Africa, a comparative study of 38 countries reported that the activities of multinational corporations are associated with differential losses in forest cover—linked to weak governance (institutions) (Assa 2018). The review clearly shows that while political, geostrategic and religious forces defined the institutional change process in Europe/North America, economic interests through multinational companies shaped institutional change in Africa. The review established that some of the significant countries with regards to cases include Australia, Ecuador, and Germany (21 + cases). In addition, Bolivia, Brazil, Cameroon, Ghana, and Canada, India registered between 8 and 14 cases, while the remaining countries registered between 1 and 7 cases (Fig. 2).

### Temporal evolution of papers and cases on forest management institutions

**Fig. 3:**
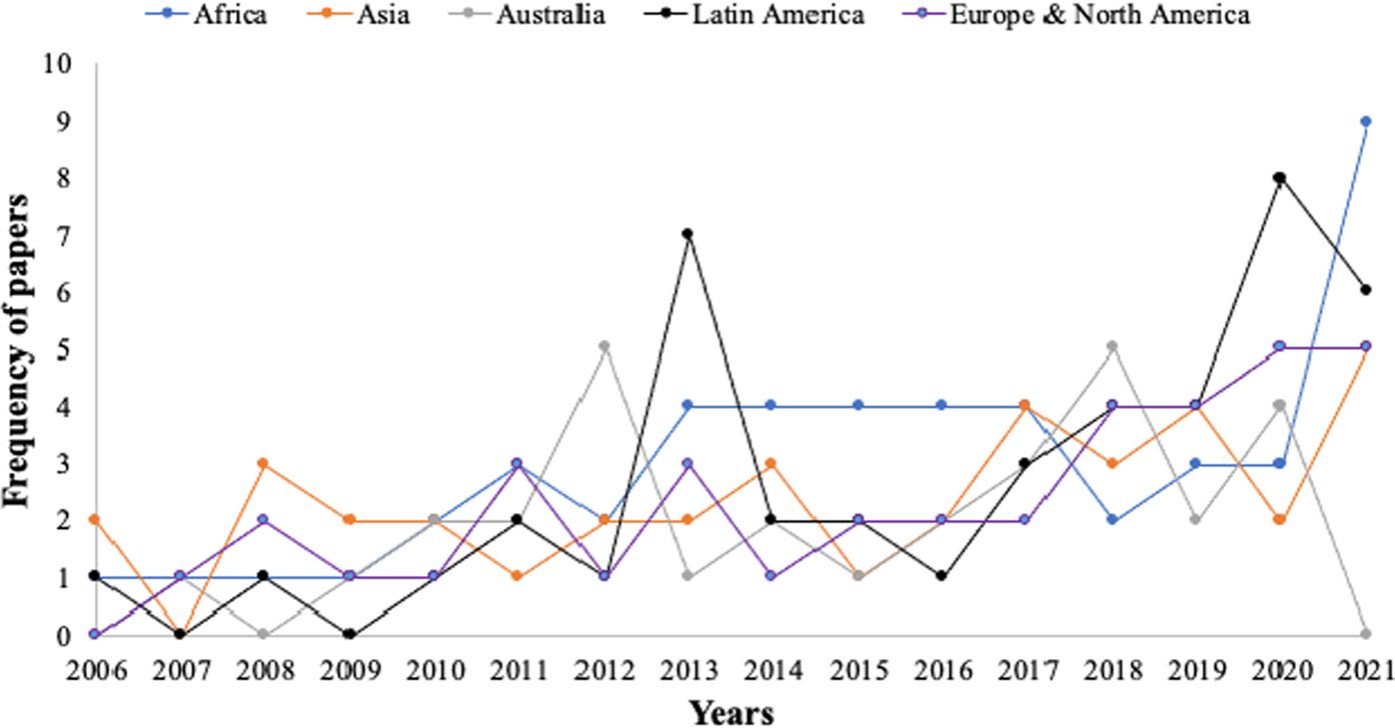
Temporal evolution of papers on forest institutions across the globe for the past 15 years

On the whole, the literature on institutions has grown over the last 15 years(Fig. 3). This could be linked to renewed interests to understand governance mishaps and to engage *in getting institutions (including FMIs) right*. In all, while the number of publications increased from 2006, the review shows that it witnessed a decline in 2009 and 2012. The growth in the literature is possibly explained by the interest to uncover institutional ‘relicts’ (in Africa) and rising environmental challenges in Latin America (bushfires and migration). Formal forest management institutions (structure and process) for forest products have received much attention in the 2000s literature. However, significant growth was observed in the literature of the 2010s, which included both formal (international) and informal (traditional and local) institutions and concepts such as ecosystem services, sustainable forest management, farm forestry, biodiversity, REDD + , and forest certification. This classification increasingly accommodated the use of endogenous and exogenous institutions, as well as state and community-based institutions. However, both classifications have been (mis)construed to represent formal and informal institutions.

### Conceptualization of forest management institutions

From a structural dimension, institutions have been most conceptualized as structures in Asia and Africa. These predominate the informal structures where Asia and Africa account for 35% and 28%, respectively, of the review’s literature reporting on informal institutional structures. Latin America closely follows them with 22%. Literature from Asia and Africa further dominates in the classification of formal institutional structures with 28% and 25%, respectively. This is closely followed by Europe/North America (23%). Asia and Africa are ‘pace-setters” in the implementation of new forest management paradigms such as community-based forest management (Kimengsi and Bhusal, 2022). The introduction of these models saw the multiplication of management structures to oversee them. This explains why the literature significantly captures the structural dimension of institutions. Process-wise, literature from Asia and Africa accounts for 48% and 30%, respectively, of the literature on informal institutions, while Australia surprisingly reports none. In the formal domain, Asia, Europe/North America, and Latin America account for over 60% of the literature reporting the formal conceptualization of institutions (Table 1).

**Table 1:** Conceptualization of forest management institutions

All studies	Institutions as structures (%)		Institutions as processes (%)	
	Formal (%)	Informal (%)	Formal (%)	Informal (%)
	71.6		88.8	
	n = 65.4	n = 30.5	n = 82.2	n = 23.4
Regions of the world				
Africa	24.8	28.3	19.8	30.4
Asia	27.9	35.0	21.6	47.9
Australia	9.3	3.3	15.4	0.0
Europe/North America	22.5	11.7	21.6	8.7
Latin America	15.5	21.7	21.6	13.0

Total number of papers (studies) = 197

Literature from Africa and Asia showed similarities in the conceptualization of institutions (Table 2); informal structures, for instance, chieftaincy and women groups, define and enforce processes (rules) which are conceived, for example, as taboos, beliefs, traditions, and customary rules. Studies in Cameroon and Burkina Faso in Africa report on bricolage manifestations involving formal and informal institutions (Kimengsi and Balgah 2021; Friman 2020), and the spatial variations in traditional institutions (Kimengsi et al. 2021, 2022b). In Europe/North America, the literature shows that informal structural institutions are conceptualized sparingly to include community leadership and inter-community forestry associations. Formally, they are reported as forest owners’ associations, political parties, protected area management, timber industry associations, resident associations, and state forest management. Informally, processes are conceived as local rules, while formally, they represent forest management policy, regulations, legal framework, local community forest governance, forest management strategies and forest marketing strategies. In Latin America, forest user groups, indigenous organizations and community management committees are frequently used in informal characterization, while labour unions, national services of protected areas, REDD + working group and Community general assemblies are used formally.

**Table 2:** Global conceptualization of forest management institutions

Regions	Institution as structures		Institution as processes	
	Informal	Formal	Formal	Informal
Africa only	Customary authorities Traditional authorities Village government Traditional priesthood Village authorities, Men’s group	Participatory forest management assoc., village natural resources committees, common initiative groups, village forest management committees, modern Media	Community-based Forest management Land tenure system Forest Act	Customary rights
Asia only	Village associations Traditional associations Traditional customary org Village committees Village conservation groups Community patrolling groups Low caste group	Forest protection committee Conservation area management committee, local forest federation Joint forest management committees, protected area authorities, forest certification agencies, village administration national trust for nature conservation, forest management committees	Community forestry guidelines Community forest operational plan International conventions State forest laws, Restoration act Regulations on land use Community forest laws National laws on social forestry Village regulations, Community forest management polices Wildlife conservation andsecurity Act	Local practices Customary regulations Endogenous rules Customary institutions
Australia only		Local/urban forest management Forest society Woods and forest department Forest municipal management	Tree farming agreement Commercial/farm forestry policy Vegetation clearing regulations Forest management plan, conventions, management act, carbon offset policy Forest Agreement	
Latin America only	Farmers’ org Forest user groups Indigenous org Community management committee	Labour unions National services of protected areas REDD + working group Community’s general assembly	Conservation laws Community-based Forest policy Forest code, Forest laws Forest tenure agreements, Decentralized environmental policy	Local land management rules
Europe/North America only	Community leadership Inter community forestry assoc	Forest owners’ assoc Political parties, protected area management, timber industry assoc. resident assoc., state forest management, conservation authorities, forest professional groups	Forest management policy Regulations Legal framework Local community forest governance Forest management strategies Forest marketing strategies	Local rules Collective rules
Africa, Asia only	Women group Chieftaincy	Village forest committee Community forest management groups		Taboos, Beliefs, Local/land customs, Traditions, informal rules, Customary rules
Africa, Asia, Australia		Community Forest User Groups/Cooperatives	Joint/collaborative/participatory forest management	
Africa, Latin America only	Ethnicity/ethnic groups	Forest management committees	Forest by-laws Forest management practices	
Africa, Europe/North America		Forest stewardship council Churches/Christianity	Institutional bricolage Forest policy framework	
Africa, Asia, Europe/North America		International conservation organization	Forest certification schemes	
Asia, Latin America	Religious groups			Values/Traditional values
Asia, Europe/North America only,	Community groups Families			
Latin America, Europe/North America	Community assembly		Protected area policy	
Australia, Europe/North America			Forest conservation policies, Forest plantation strategies or subsidy	
Africa, Asia, Latin America		Civil Society Organizations	Payment for ecosystem services REDD +	
Africa, Asia, Latin America, Europe/North America				Local/social/moral/community norms
Asia, Australia Latin America, Europe/North America only		Forest industries/companies or Private sector		
All regions	Villages councils	NGOs, Forestry department/commission Central government/State Forest management institutions	State forest/land policies National/State Forest rules/regulations	

Local land management rules constitute the key informal process in Latin America, while conservation laws, community-based forest policy, forest codes, forest laws, forest tenure agreements, and decentralized environmental policy appear in the formal conception of institutions. On the whole, a more diverse conceptualization of institutions (structures and processes) appear in the literature from Africa and Asia, followed by Latin America. The diversity is rooted in the diverse ethnic arrangements which characterize these regions. Africa, is the most ethnically diverse region in the world (Fearon 2003). This diversity accounts for the diversity in the nomenclature employed for forest management institutions—leading to the diversity in their conceptualization. With more empirical cases emanating from these settings, it is plausible to suggest that more in-depth and varied analysis about forest management institutions has been explored in these settings. Furthermore, the plethora of governance challenges in the management of natural resources (forest in this case) which is associated with such settings further explains the multifarious typification of institutions. In another dimension, structures and processes are surprisingly only conceptualized formally in the context of Australia—suggesting a significant drift away from informality to the pursuit of more formal, state-sanctioned institutions.

### Compliance with forest management institutions

**Fig. 4:**
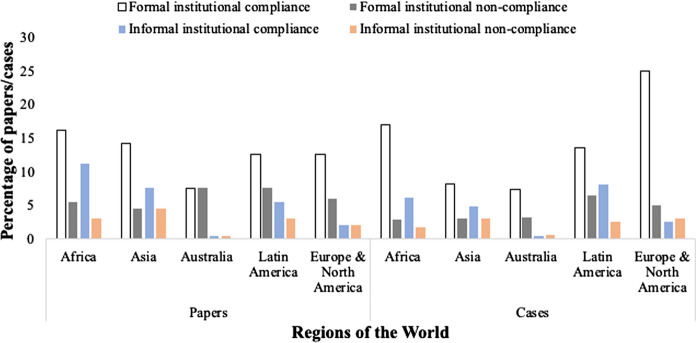
Global statistics of institutional compliance and non-compliance by regions. Left chart—papers (*N* = 197, n for compliance = 133, *n* for non-compliance = 64, Right chart—Cases (*N* = 491, n for compliance = 362, *n* for non-compliance = 129). *Note* Some papers reported compliance/compliance to formal and informal institutions simultaneously

From the review, Africa, Asia, and Latin America report the highest cases of compliance in the informal institutional set-up. These settings have had a history linked to traditional institutions which were made to interact with colonially shaped institutions during their history. However, some degree of closeness to cultural institutions could be reported for these regions. The existence of compliance in the literature for Africa, Asia and Latin America is enough pointer to the multiplicity of institutional structures and processes which require monitoring against (non)compliance. Additionally, the interaction between formal and informal institutions, including the fallouts of colonial influence, led to the multiplicity of institutions. This possibly explains why compliance predominates the literature in the three regions. In Africa, for instance, pre-colonial types of resource use included the royal hunting preserves of the amaZulu and amaSwati people, and the *kgotla* system of land management practiced by the Batswana people (Ghai 1992; Fabricius 2004). Further, the making of access and use rules for natural resources in Mali (Moorehead 1989) and Botswana (Ostrom 1990), all indicate how endogenous cultural institutions shaped forest use. Likewise, Khasi, Garo and Jaintia tribes in Meghalaya of India, and traditional customary organization “Lembaga Adat” in Indonesia have not only conserved forest resources but also ensured its capacity to deliver ecosystem goods and services in sustainable manner (Mehring et al. 2011; Tiwari et al. 2013). Europe/North America registered few studies on informal institutional compliance, while this was non-existent for Australia—apparently due to the non-reported case of informal arrangements. Regarding non-compliance, articles from Latin America reported the highest case of non-compliance. This could be explained by the progressive decline in the informal institutions due to globalization and market forces which seemed to have permeated communities around the Amazon (Blundo-Canto et al. 2020). In the formal domain, non-compliance was significantly registered for Europe/North America, Africa, and Latin America (Fig. 4).

It is important to note that some of the evoked reasons behind (non)compliance still require further investigation. For example, significant contextual variations in peoples’ attitudes and adherence to forest-sector institutions and governance in Asia, Africa, Latin America, and North America are directly linked to the disparities of key underlying and broader factors such as institutional models and policy frameworks for decentralization (Shackleton et al. 2002; Ribot 2003; Larson 2012; Mustalahti et al. 2020). Thus, a broader focus on institutional factors—rather than isolated reasons—is important to sufficiently explain (non-)compliance dynamics.

### Forest management Institutional compliance determinants

From the analysis, political and economic factors were recurrent in the literature as key forces that influence institutional compliance. For instance, in Europe/North America, Latin America and Australia, political factors significantly influenced compliance (Table 3). Some of these key determinants include conflicts, policy enforcement, power relations and governance structure (Latin America) and actor network, policy development, and governance structure (Europe/North America). Politics and geostrategy contributed to defining natural resource (forest in this case) institutions in Europe and North America. However, in Latin America, the rise in challenges linked to migration and bush fires also stand as key determinants of forest management institutional compliance. Economic factors in Europe/North America and Africa determined compliance. In Africa, economic factors linked to private enterprises and market-based mechanisms significantly featured in the literature as determinants of institutional compliance. For instance, aspects linked to donor income/investment and material aid, community forest expenditure and benefits, poverty were common. In Europe/North America, the economic viability of forest land use, forest certification, amongst others, were reported in the articles. In Nigeria, economic incentives (incomes) from farming activities, NTFPs use and non-traditional employment shaped compliance (Ezebilo 2011). On the whole, ecological, socio-cultural and demographic factors did not significantly explain compliance with forest management institutions. The case of ecological determinants in surprising given the litany of ecological campaigns which have been introduced in Africa (e.g., Leventon et al. 2014; Senganimalunje et al. 2016), Asia (Gilani et al. 2017) and Latin America (Entenmann and Schmitt 2013; Kowler et al. 2020) for instance, to foster conservation. Furthermore, socio-cultural diversity as viewed in Africa warrants some diversity in the way people adhere to institutions—both formal and informal. In Tanzania, trust in institutions was a significant predictor of participation intensity of the households in forest management (Luswaga and Nuppenau 2020). However, in Europe, differences in the attitudes of actors with regard to pursuing sustainable development significantly shaped compliance with forest management institutions (Jankovska et al. 2010).

**Table 3:** Forest management institutional compliance determinants to by regions

Inst. determinants	No. of cases per region					
	Africa	Asia	Australia	Latin America	Europe and North America	Total
Ecological	3	0	7	14	1	25
Economic	54	2	8	17	57	138
Political	23	17	50	56	83	229
Socio-cultural	9	9	0	5	3	26
Demographic	0	1	0	0	0	1
Multi-factors	15	27	13	13	4	72

### Forest management institutional outcomes

The review indicates that political outcomes were the most significant for Europe/North America, followed by Latin America and Australia (Table 4). Some key political outcomes included policy fragmentation, market formation failures, and reduced legitimacy of FSC certification (Europe/North America). This is understandable, considering that political and geostrategic forces were key determinants of institutional compliance. In Latin America,the setting up of provincial regulations which undermine enforcement of forest regime, rule breaking, challenges with the day-to-day operational institutions, inequitable benefit-sharing mechanism; the absence of law enforcement on sustainability of and access to non-wood forest products were common. Rule breaking is potentially triggered by increasing in-migration and the upsurge of bushfires. Bottazzi et al. (2014) showed how incentive-based systems of institutions facilitated the allocation and use of funds in REDD + programmes. In all these, deforestation persisted in the midst of lost and/or bypassed institutions (Carvalho et al. 2019). In Australia, divergent views characterized the seeking of solutions to enhance inter-departmental and inter-municipal coordination (Ordóñez et al. 2020). Positive ecological outcomes were significantly reported for Africa (forest or biodiversity protection/conservation, improved forest condition and surface water quality, sustainable forest or ecosystem management, planting of timber and fruit trees) and Latin America (fostering forest conservation, stabilization and/or decrease of deforestation, sustainable forest management). Furthermore, Europe/North America, Africa and Asia respectively reported positive economic outcomes linked to the generation of net monetary gains from parks, and from the wood harvesting and marketing (Europe/North America), higher incomes derived from certification, profits derived under community forestry, and the augmentation of household cash income (Africa). In Asia, studies report the positive outcomes linked to the forests’ substantial contribution to local livelihoods and income (Muhammed et al. 2008; Harada and Wiyono 2014; Barnes and van Laerhoven 2015).

**Table 4:** Forest management institutional outcomes by regions

Institutional outcomes	No. of cases per region					Total
	Africa	Asia	Australia	Europe/North America	Latin America	
Ecological ( +)	61	19	8	3	37	129
Ecological (−)	5	5	15	2	10	37
Economic ( +)	11	7	4	49	4	75
Economic (−)	3	4	1	0	14	22
Political	16	25	26	70	37	174
Socio-cultural	9	13	24	22	9	77

Some case studies have multiple institutional outcomes

Australia witnessed the most negative ecological outcomes. For instance, regional forest agreements were characterized by poor governance, leading to failures in biodiversity protection and ecosystem maintenance. This further precipitated the over-commitment of forest resources to wood production (Lindenmayer 2018). In Latin America, significant deforestation was observed for the Guarayos Indigenous Territory from 2000 to 2017—primarily driven by agricultural commodity production (He et al. 2019), while in Africa, Garekae et al. (2020) reported forest and wildlife decline in Botswana, linked to sectoral bias. Furthermore, the articles reported significant negative economic outcomes for Latin America, Asia, and Africa. Some of the reported outcomes include financial resource decline (Latin America), the timber-centric and market-oriented nature of community forests (Asia) (Bhusal et al. 2020), and the manifestations of elite capture in Africa. In Malawi, for instance, co-management programmes did not lead to positive outcomes, i.e., community organization, forest access, forest product availability and commercialization of forest products (Senganimalunje et al. 2016). On the whole socio-cultural outcomes were prevalent in Australia, Europe/North America, and Asia. Here, reported issues were linked to public acceptance of plantation policy, the improvement in communication of forest owners' associations and increased reliance on informal relationships. A case in point is linked to forest policy in Australia which led to several negative social impacts, including uncertainty, perceived injustice, and financial stress (Loxton et al. 2014). In the case of Asia, it was linked to inequalities among local actors, demographic changes and transformations in local social structures, gender inequality, successful collaboration between NGOs and community-based organizations**,** conflicts between communities and state forest enterprises (Adhikari and Lovett 2006; Barnes and van Laerhoven 2015).

### Methodological approaches

**Fig. 5:**
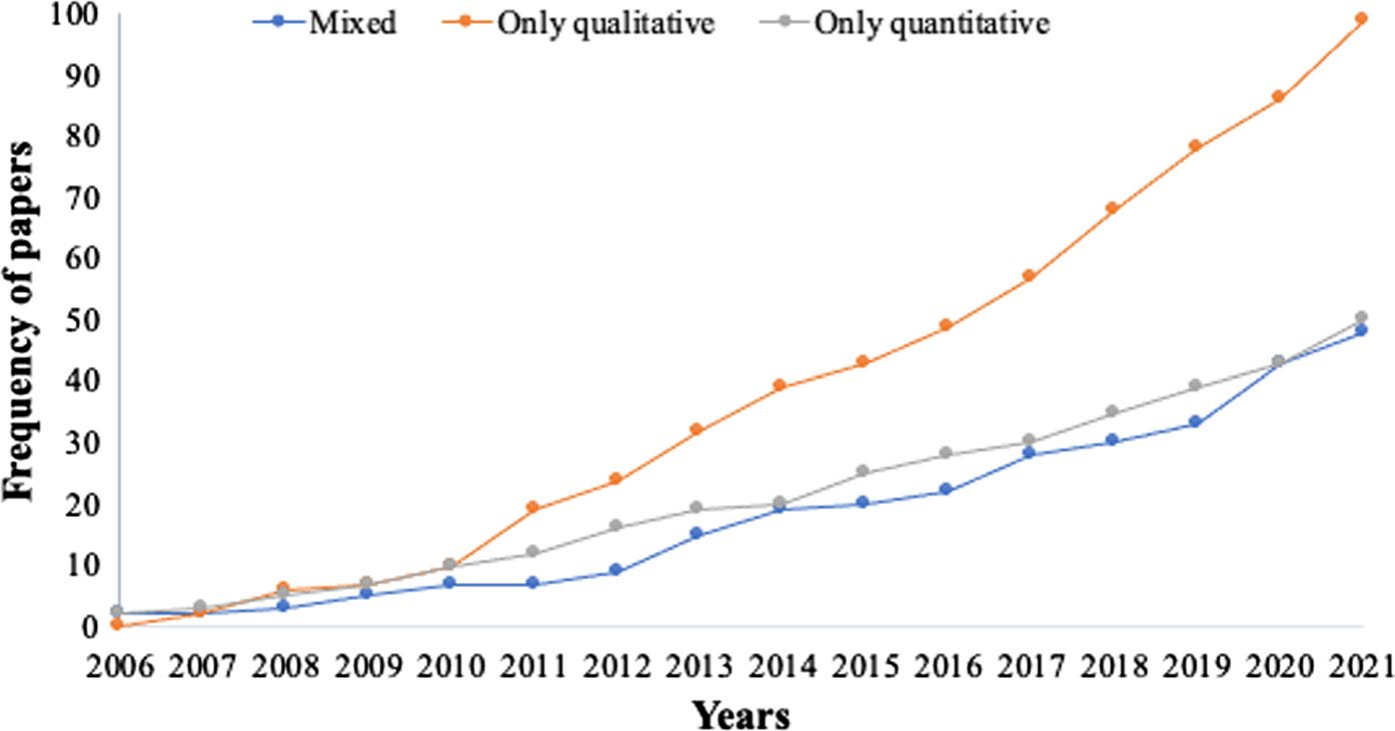
Cumulative distribution of paper and adoption of methods

The analysis reveals that globally mixed methods approaches have been least prioritized in the study of forest management institutions. For instance, between 2006 and 2021, we observed a growing trend in the application of qualitative methods; only 2 articles were reported in 2007, while this peaked to 99 in 2021. However, the use of quantitative and mixed methods approaches was significantly lower (Fig. 5). Considering the intricacies linked to the study of institutions, the prioritization of qualitative approaches is understandable. However, with growing interest in employing more robust data collection and analysis methods, it is only germane to report that studies have not prioritized mixed methods approaches so far (Malina et al. 2011; Karolina et al. 2021).

A slight increase in the application of mixed methods approaches is observed (Table 5) from 22% between 2006 and 2010 to 26% between 2016 and 2021, while there was a progressive decline in the sole application of quantitative methods from 37 to 23% within this time period. A slight decrease is also observed for qualitative methods, from 54 to 51% between 2011 and 2021.

**Table 5:** Methods employed over time in the study of forest management institutions (% in parenthesis)

Duration	Mixed	Only qualitative	Only quantitative	Total
2006–2010	6 (22.22)	11(40.74)	10 (37.04)	27
2011–2015	13 (21.31)	33 (54.10)	15 (24.59)	61
2016–2021	28 (25.69)	56 (51.38)	25 (22.94)	109
Grand Total	47 (23.86)	100 (50.76)	50 (25.38)	197

On the whole, while the use of only qualitative methods in the study of forest management institutions increased over time, the adoption of only quantitative approaches witnessed a decrease (Table 5). Studies in Africa have largely prioritized the sole application of quantitative methods, as 42% of the papers reported this approach (Table 6), while mixed methods (25%) were least prioritized. This could be linked to the growing ‘quantification revolution’ in research across the region. While quantitative analysis provides some pointers to institutional questions, they hide significant intricacies which could be revealed by solely qualitative or, better still, mixed methods analytical approaches. In Latin America, however, a significant proportion of the studies (40%) employed solely qualitative methods, followed by mixed methods (35%) and then quantitative methods (26%). Likewise, the highest proportion (58%) of studies draw from qualitative methods in Asia and Australia, whereas mixed-methods were prioritized as the second highest (37%) in Asia and the least (10%) in Australia (Table 6).

**Table 6:** Methods used by different continents (percentage in parenthesis)

Continents	Mixed	Only qualitative	Only quantitative	Grand total (% in parenthesis)
Africa	12 (25)	16 (33)	20 (42)	48 (100)
Latin America	15 (35)	17 (40)	11 (26)	43 (100)
Asia	14 (37)	22 (58)	2 (5)	38 (100)
Australia	3 (10)	18 (58)	10 (32)	31 (100)
Europe and North America	3 (8)	27 (73)	7 (19)	37 (100)
Grand total	47 (24)	100 (51)	50 (25)	197 (100)

On the whole, while studies in Africa employed more of only quantitative methods over qualitative ones, research on forest management institutions in Europe and North America prioritized only qualitative methods over only quantitative ones. In North America/Europe, 73% of the studies employed the qualitative approach, followed by quantitative (19%). The least employed approach is mixed-methods, as only 8% used this approach (Table 6). Overall, qualitative methods have been significantly employed globally except in Africa, while mixed methods were the least adopted in all regions except Asia and Latin America.

### Perspectives on the conceptual and methodological advancement of research on FMIs

The literature so far presents a fragmented conceptualization of forest management institutions. For instance, institutions are broadly categorized as formal or informal on the one hand and as exogenous and endogenous on the other hand. This is based on the premise that not all endogenous institutions are informal institutions. A more detailed conceptualization which captures the formal and informal dimension, including the endogenous and exogenous categorization, is helpful to advance theoretical developments in the field of institutions in relation to forest management. Additionally, institutions seem to exhibit stream-like attributes; an approach which further conceptualizes them as ephemeral (very short-term arrangements made by forest actors to facilitate forest resource use conflict minimization), *partially enduring (*arrangements that temporarily become a norm but fizzle out as new actors take over (Kimengsi et al. 2022b); and *enduring (*institutions are either codified (formal) and/or take the status of customs and values which transcend several generations (Ostrom 1990). In both cases, empirical studies geared towards establishing these proposed conceptual approaches are needed. Future research also needs to advance the “marriage” between actors and institutions.

From a methodological standpoint, further studies should prioritize methods based reviews (Palmatier et al. 2018) to enable researchers synthesize in detail, the design and instruments used so far, the approaches employed in data collection approaches and *pros* and *cons* linked to the methods employed. This will further inform the application of methodological approaches and instruments for future empirical studies on forest management institutions. Also, multi-country studies, employing mixed-methods approaches are needed to analyze institutions in forest use and management.

## Review Limitations

This review provides an initial synthesis of the literature on forest management institutions from a global perspective. It is helpful in the identification of region-specific research needs in the ever-evolving field of institutions. A couple of limitations could be raised: Firstly, the conceptual analysis of institutions does not incorporate the exogenous versus endogenous dichotomy. With growing interest to further explore the typology and source of institutions, including whether management outcomes are a function of more endogenous or exogenous institutional arrangements (Kimengsi et al. 2022b), future reviews and empirical studies should incorporate this dimension. Secondly, the regional clustering of institutions might shade details linked to how institutions are conceptualized and the outcomes they effectively produce. Although case studies are used, it is not possible in a single review to derive all these conceptual details, which might vary even within regions. Taking Africa, for instance, diversity in the region’s culture requires country-specific analysis of institutions. Latin America’s diversity precipitates ‘institutional shopping’ (Wartmann et al. 2016 Thirdly, institutions do not operate in isolation—therefore an actor-centred/power dimension is required to better appreciate institutional arrangements (Giessen et al. 2014; Ongolo et al. 2021). Therefore, a review of the actor and power dimensions of institutions is required to inform subsequent empirical studies. Fourthly, while the paper reports on compliance, the level of compliance is not reported, and the factors which militate for or against compliance. Fifthly, we selected articles which were exclusively published in English language and indexed in certain data bases. In doing so, we ignored papers which might have been published in French, Spanish, Amharic, Kiswahili, Nepali and other languages; such articles might have provided further compelling details on the region-specific dynamics of forest management institutions. We call for subsequent reviews to aim at valorizing such studies.

The current socio-ecological outcomes linked to the upsurge of pandemics (e.g. COVID-19) further justify the need to pay more attention to the management of forests and forest resources (Tollefson 2020; Saxena et al. 2021). These details, which vary over space and time, and may potentially assume a different dimension under the current COVID-19 scenario (Saxena et al. 2021), require extensive review and further empirical grounding. When pandemic prevention hinges on forest management to some extent, it is imperative to further explore the role of FMIs. Further reviews could emphasize the extent of compliance and the conditions under which (non)compliance prevails in the context of pandemics. Additionally, institutional change which is triggered by health crises (e.g., pandemics) still needs to be further established.

Finally, our review of the methods focused on providing a snapshot of the approaches, following the broad categorization of qualitative, quantitative and methods. This does not provide details on the specific qualitative methods employed (e.g., key informant interviews, participant observations, vignettes, focus group discussions). Future methods-based reviews should consider these.

## Conclusion

To define conceptual and methodological pathways for future studies on forest management institutions (FMIs), this study undertakes a systematic review of the literature on FMIs using 197 papers (491 cases). From the study, the following conclusions are plausible: *Firstly, while* forest management institutions literature has witnessed a growth, this is most significant in Africa and Latin America. *Secondly,* the structure-process conceptualization of institutions (formal and informal) predominates in Asia and Africa. Process-wise, studies from Australia surprisingly did not report on a single process-linked institution. This merits further studies which pays attention to the identification of such institutions. The literature also reports on the drift away from informality to the pursuit of more formal, state-sanctioned institutional arrangements in Australia. *Thirdly*, global south regions—Africa, Asia, and Latin America—report the highest cases of compliance in the informal institutional set-up, while non-compliance was significantly registered for Europe/North America in the formal domain. *Fourthly*, politico-economic factors significantly influence institutional compliance in Europe/North America, while economic factors shape compliance in Africa. On the whole, ecological, socio-cultural, and demographic factors were reported to less significantly explain compliance with forest management institutions (FMIs). *Fifthly*, while forest management institutions in Europe/North America significantly contributed to determining politico-economic outcomes, those in Africa and Latin America contributed to positive ecological and negative economic outcomes. *Finally,* mixed methods approaches have been least prioritized in the study of forest management institutions; in Africa, the sole application of quantitative methods was prioritized. Future research needs to (1) extend the conceptualization of institutions, (2) increase multi-case and multi-country studies on FMIs especially for Asia and Australia, (3) empirically ground informality in the institutional set up of forest management in Australia, (4) establish in detail, the extent of (non)compliance, their spatio-temporal variations, and determinants, and (5) valorize the application of mixed-methods approaches in the study of FMIs across the globe.

## Supplementary Information

Below is the link to the electronic supplementary material.

Supplementary file1 (XLSX 108 kb)

Supplementary file1 (DOCX 12 kb)
