# Supplementary material for: A global systematic review of forest management institutions: towards a new research agenda
Source: Landsc Ecol. 2022 Dec 24;38(2):307–26. doi: 10.1007/s10980-022-01577-8 (PMC9789374; doi:10.1007/s10980-022-01577-8)
Supplement: Supplementary file 2 — Supplementary file1 (DOCX 12 kb) [file 10980_2022_1577_MOESM2_ESM.docx]

**Appendix**

| **Institutional Parameters** | **Sub-institutional parameters** | **Search combinations** |
| --- | --- | --- |
| Institutions as structures (informal) | Village forest management committees Traditional councils Village councils  Community based institutions village leadership  Customary authorities  Chieftaincy  Local gods or deities | Forest or Forests and institutions or “Village Forest management committees” or “Traditional councils” or “Village councils” or “Community based institutions” or “village leadership” or “Customary authorities” Chieftaincy or “local gods” or deities and “Latin America” or “Central America” or “South America” or Caribbean or Europe or Asia or Africa or “North America” or Australia |
| Institutions as structures (formal) | Civil Society Organizations (CSOs) Community Based Organizations (CBOs), Cooperatives, NGOs State Organizations, Government Organizations, Ministries District Forest officials | Forest or Forests and institutions or management or “Civil Society Organizations” or “Community Based Organizations” or Cooperatives or NGOs or “State Organizations” or “Government Organizations” or Ministries or “District Forest officials” and “Latin America” or “Central America” or “South America” or Caribbean or Europe or Asia or Africa or “North America” or Australia |
| Institutions as processes (formal) | Institutions, Legal framework Exogenous institutions, international agreements, Policies Laws, Institutional bricolage  Governance, Institutional change Hybrid institutions, Decentralization | Forest or Forests and institutions or “Legal framework” or “Exogenous institutions” or “International agreements” or conventions or Policies or laws or “institutional bricolage” or governance or “institutional change” or “hybrid institutions” or decentralization and “Latin America” or “Central America” or “South America” or Caribbean or Europe or Asia or Africa or “North America” or Australia |
| Institutions as processes (informal) | Endogenous institutions Cultural institutions Indigenous practices Informal institutions Traditional institutions Customs, Rules, Traditions, Beliefs Norms, Values, Taboos | Forest or Forests and institutions or “Endogenous institutions” or “Cultural institutions” or “Indigenous practices” or “Informal institutions” or “Traditional institutions” or customs or rules or traditions or beliefs or norms or values or taboos and “Latin America” or “Central America” or “South America” or Caribbean or Europe or Asia or Africa or “North America” or Australia |
